# Linear fine-tuning: a linear transformation based transfer strategy for deep MRI reconstruction

**DOI:** 10.3389/fnins.2023.1202143

**Published:** 2023-06-20

**Authors:** Wanqing Bi, Jianan Xv, Mengdie Song, Xiaohan Hao, Dayong Gao, Fulang Qi

**Affiliations:** ^1^The Centers for Biomedical Engineering, University of Science and Technology of China, Hefei, Anhui, China; ^2^Fuqing Medical Co., Ltd., Hefei, Anhui, China; ^3^Department of Mechanical Engineering, University of Washington, Seattle, WA, United States

**Keywords:** magnetic resonance imaging reconstruction, deep learning, transfer learning, fine-tuning, transfer strategy

## Abstract

**Introduction:**

Fine-tuning (FT) is a generally adopted transfer learning method for deep learning-based magnetic resonance imaging (MRI) reconstruction. In this approach, the reconstruction model is initialized with pre-trained weights derived from a source domain with ample data and subsequently updated with limited data from the target domain. However, the direct full-weight update strategy can pose the risk of "catastrophic forgetting" and overfitting, hindering its effectiveness. The goal of this study is to develop a zero-weight update transfer strategy to preserve pre-trained generic knowledge and reduce overfitting.

**Methods:**

Based on the commonality between the source and target domains, we assume a linear transformation relationship of the optimal model weights from the source domain to the target domain. Accordingly, we propose a novel transfer strategy, linear fine-tuning (LFT), which introduces scaling and shifting (SS) factors into the pre-trained model. In contrast to FT, LFT only updates SS factors in the transfer phase, while the pre-trained weights remain fixed.

**Results:**

To evaluate the proposed LFT, we designed three different transfer scenarios and conducted a comparative analysis of FT, LFT, and other methods at various sampling rates and data volumes. In the transfer scenario between different contrasts, LFT outperforms typical transfer strategies at various sampling rates and considerably reduces artifacts on reconstructed images. In transfer scenarios between different slice directions or anatomical structures, LFT surpasses the FT method, particularly when the target domain contains a decreasing number of training images, with a maximum improvement of up to 2.06 dB (5.89%) in peak signal-to-noise ratio.

**Discussion:**

The LFT strategy shows great potential to address the issues of "catastrophic forgetting" and overfitting in transfer scenarios for MRI reconstruction, while reducing the reliance on the amount of data in the target domain. Linear fine-tuning is expected to shorten the development cycle of reconstruction models for adapting complicated clinical scenarios, thereby enhancing the clinical applicability of deep MRI reconstruction.

## 1. Introduction

Magnetic resonance imaging (MRI) includes diverse sequences that provide distinct types of anatomical and pathological information, catering to a wide range of clinical needs (Yousaf et al., 2018). For instance, T1-weighted spin-echo sequences can be employed to measure the cross-sectional area of visceral and subcutaneous fat in the abdomen (Lancaster et al., 1991), as T1-weighted images provide the most anatomically-relevant details. Additionally, T2-weighted images highlight lesions and can be used to identify myocardial edema (Eitel and Friedrich, 2011), determine the area at risk in non-reperfused infarction (Aletras et al., 2006), etc. As a downside, standard scan sequences used in clinical routines generally require a long imaging time, which can aggravate patient discomfort and introduce severe motion artifacts. In an effort to speed up the acquisition of MRI, reconstruction techniques (Roy and Kailath, 1989; Pruessmann et al., 1999; Griswold et al., 2002; Block et al., 2007; Hamilton et al., 2017) based on under-sampled k-space data have been developed, albeit at the cost of increased hardware requirements or lengthened reconstruction time.

Recently, deep learning-based technologies have gained much attention owing to the powerful image representation capabilities and fast image generation speed (Lee et al., 2018; Quan et al., 2018; Hosseini et al., 2020; Cole et al., 2021). Numerous neural network frameworks have been proposed for MRI reconstruction, establishing an end-to-end non-linear mapping from under-sampled data to fully-sampled images. Wang et al. (2016) introduced a convolutional neural network (CNN) that employed a large number of MR images as training datasets to recover the delicate structures and details of test data. Additionally, Quan et al. (2018) designed a variant of a fully-residual convolutional auto-encoder and generative adversarial networks (GAN) called RefineGAN, which incorporated a cyclic data consistency loss and a chained architecture to enhance the reconstruction quality. Despite being a powerful tool for MRI reconstruction, deep learning still suffers from a notable inherent drawback: limited generalizability (Gavrikov and Keuper, 2022). It was found that deep learning algorithms are sensitive to shifts in the distribution of input data (Knoll et al., 2019; Antun et al., 2020). Knoll et al. (2019) summarized the deviations between training and testing led to substantial decreases in reconstruction image quality. Antun et al. (2020) demonstrated that even some certain tiny, almost imperceptible perturbations may result in severe artifacts in the reconstruction. Moreover, even for one specific anatomical part, the reconstructed MR images exhibit obvious stylistic variations when different sequences or different scan parameters are performed. Therefore, adapting a reconstruction model to diverse clinical scenarios is challenging.

To enhance the generalization ability of the neural networks, a straightforward approach is to collect ample data encompassing various clinical scenarios. However, medical imaging domains generally face a dearth of data, which is both time-consuming and expensive to acquire. Alternatively, transfer learning strategies (Tajbakhsh et al., 2016) have been developed, focusing on how to transfer knowledge from the source domain to the target domain (Romero et al., 2020). Since an explicit paradigm for designing transfer strategies is not available, multiple factors such as the task attribute, the total amount of data, and the amount of labeled data in the source and target domains should be considered comprehensively. Kamphenkel et al. (2018) proposed an unsupervised domain adaptation method for breast cancer classification to transform the target data to the source domain without any label information of target data. Faced with the inability to obtain paired images of the same subject, Zhang et al. (2022) designed a novel unsupervised domain adaptation approach for tackling the distribution discrepancy across domains in medical image segmentation.

Regarding the MRI reconstruction scenario involved in this study, it is characterized by the following aspects. Firstly, the tasks in both the source and target domains are identical, i.e., reconstructing under-sampled aliased images into aliasing-free images. Secondly, the source domain is composed of a large-scale public dataset, while the target domain has a relatively small data volume. Thirdly, network training usually takes full-sampling data as the reference without requiring additional labeling. Hence, in this case, fine-tuning (FT) (Pan and Yang, 2010; Dar et al., 2020; Frégier and Gouray, 2021) is the most commonly used transfer learning method that updates pre-trained models to target domains. The FT strategy leverages pre-trained weights as initialization and relearns all the weights of convolutional layers in the transfer phase, which has been investigated in many studies. Dar et al. (2020) pre-trained networks on large public MR datasets and then fine-tuned them using only tens of brain MR images from the target domain, achieving performance comparable to networks trained directly on thousands of target images. Arshad et al. (2021) obtained satisfactory reconstruction of MR images based on a pre-trained U-Net through end-to-end FT under various magnetic field strengths, anatomies, and acceleration factors. Lv et al. (2021a) focused on the generalization ability of the multi-channel MRI reconstruction network and highlighted the critical role of FT in adapting to a particular target application using only a few training cases.

All of the aforementioned research has employed FT to achieve reliable reconstructions of under-sampled images in different transfer scenarios, demonstrating the potential of knowledge transfer for reconstruction. However, two major limitations of FT hinder its effectiveness. Firstly, updating all the pre-trained weights implies that the target model may forget the pre-trained knowledge when adapting to a new task, known as “catastrophic forgetting” (Lopez-Paz and Ranzato, 2017). This can be improved as distinct MR images exhibit many similar features despite variations in contrast, slicing direction, and anatomy. Additionally, networks mostly contain a mass of weights, and the full-weight adjustment on a small target dataset may result in overfitting (Sun et al., 2019). Recently, layer-wise FT (Tajbakhsh et al., 2016; Amiri et al., 2020), a transfer strategy of FT, has been applied in the field of medical imaging for transfer learning. Layer-wise FT freezes the weights of specific layers and tunes the rest in the transfer phase. Although layer-wise FT has the potential to alleviate “catastrophic forgetting” by reducing the degree of weight updates, the determination of an optimal freezing strategy is scenario-dependent, making it difficult to design a general scheme for all transfer scenarios as in the case of FT. Therefore, layer-wise FT is rarely applied in the transfer learning of deep MRI reconstruction.

In this study, we propose a novel transfer strategy of FT, termed linear fine-tuning (LFT), which assists in effective transfer and is applicable to MRI reconstruction. The approach assumes a linear transformation relationship between the source and target models. Specifically, networks pre-trained on a large-scale source dataset can be transferred to the target domain by adjusting the scaling and shifting (*SS*) factors. These two factors are integrated into the *SS* block in a learnable fashion, which is merged with regular convolution to adjust for linear deviation. Unlike FT, LFT avoids changing the entire pre-trained weights and instead updates the *SS* factors continuously while freezing the pre-trained weights and biases. The proposed zero-weight strategy benefits learning specific knowledge while avoiding forgetting the generic knowledge by fixing the pre-trained weights. Additionally, LFT entails a considerably smaller number of weights to be adjusted, thereby reducing the risk of overfitting.

Our contributions can be summarized as follows.

- We hypothesize that advanced features of different MR images can be represented as different linear combinations of the same basic features.
- Based on the proposed assumption, the LFT strategy introduces two coefficients, namely *SS* factors, in convolutional layers for the linear transformation of features. In the transfer phase, only the *SS* factors are updated, thereby mitigating the risk of “catastrophic forgetting” and overfitting.
- We conducted extensive experiments on various MR datasets with different data volumes and sampling rates. The results suggest that the proposed LFT strategy generally outperforms other methods and achieves high-quality reconstruction, especially in the case of small samples.

## 2. Methods and materials

### 2.1. Preliminary

#### 2.1.1. Definitions and notations

Since the key part of the LFT strategy implementation is the *SS* factors inserted into the convolutional layer, some concepts are defined to facilitate the explanation. In general, one of the fundamental components of a neural network is the convolutional layer, which acts as a feature extractor. The two-dimensional convolutional layer transforms an input tensor *U* of size *M* × *H* × *W* into an output tensor *V* of size *N* × *H* × *W* by applying *N* convolution filters *F*^(1)^, *F*^(2)^, ⋯ , *F*^(*N*)^. The feature maps generated by each filter are defined as advanced features *V*_*a*_, such that


(1) $$\begin{array}{l} V_{a}=U*F, \end{array}$$

where *F* ∈ {*F*^(1)^, *F*^(2)^, ⋯ , *F*^(*N*)^}, * represents the multichannel convolution operator, and *V*_*a*_ of each filter corresponds to one channel of *V*.

Each filter *F* contains *M* kernels, namely *K*_(1)_, *K*_(2)_, ⋯ , *K*_(*M*)_, with weights *W*. The output of each kernel is defined as basic features *V*_*b*_, via


(2) $$\begin{array}{l} V_{b\left(m\right)}=U_{\left(m\right)}⊙W_{\left(m\right)}+b_{\left(m\right)}, \end{array}$$

where *K*_(*m*)_ ∈ {*K*_(1)_, *K*_(2)_, ⋯ , *K*_(*M*)_}, with weight *W*_(*m*)_. *U*_(*m*)_ indicates the channel of *U* corresponding to *K*_(*m*)_. ⊙ denotes the convolution operator and *b* is the bias of the convolutional layer.

Equations (1) and (2) are combined to obtain


(3) $$\begin{array}{l} V_{a}=\overset{M}{\underset{m=1}{\sum }}V_{b\left(m\right)}=\overset{M}{\underset{m=1}{\sum }}\left(U_{\left(m\right)}⊙W_{\left(m\right)}+b_{\left(m\right)}\right). \end{array}$$

**Figure 1:**
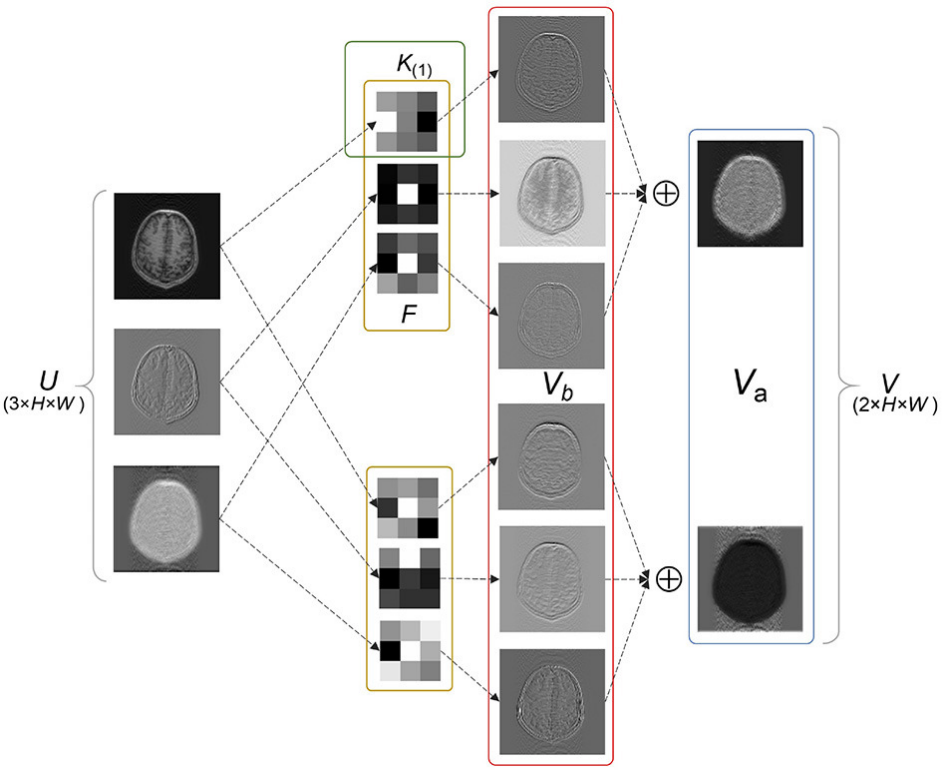
Exemplary basic and advanced features. *V*_*b*_ and *V*_*a*_ represent the outputs of each kernel and each filter, respectively. *V*_*a*_ shows two types of advanced features including in-contour artifacts **(top)** and out-of-contour artifacts **(bottom)**.

Equation (3) indicates the convolution operation can be split into two parts: feature extraction and linear combination. Specifically, as illustrated in Figure 1, an example of a filter *F* containing two kernels *K*_(1)_, *K*_(2)_ is used. Each kernel slides over the corresponding channel of *U* and extracts basic features. Subsequently, the extracted features from different kernels are combined into advanced features, which are commonly described as a feature map. Advanced features typically contain special significance that reflects the function of the filter, especially in the deep layers of the network. Figure 1 shows two types of advanced features, including in-contour artifacts (top) and out-of-contour artifacts (bottom).

#### 2.1.2. Fine-tuning

**Figure 2:**
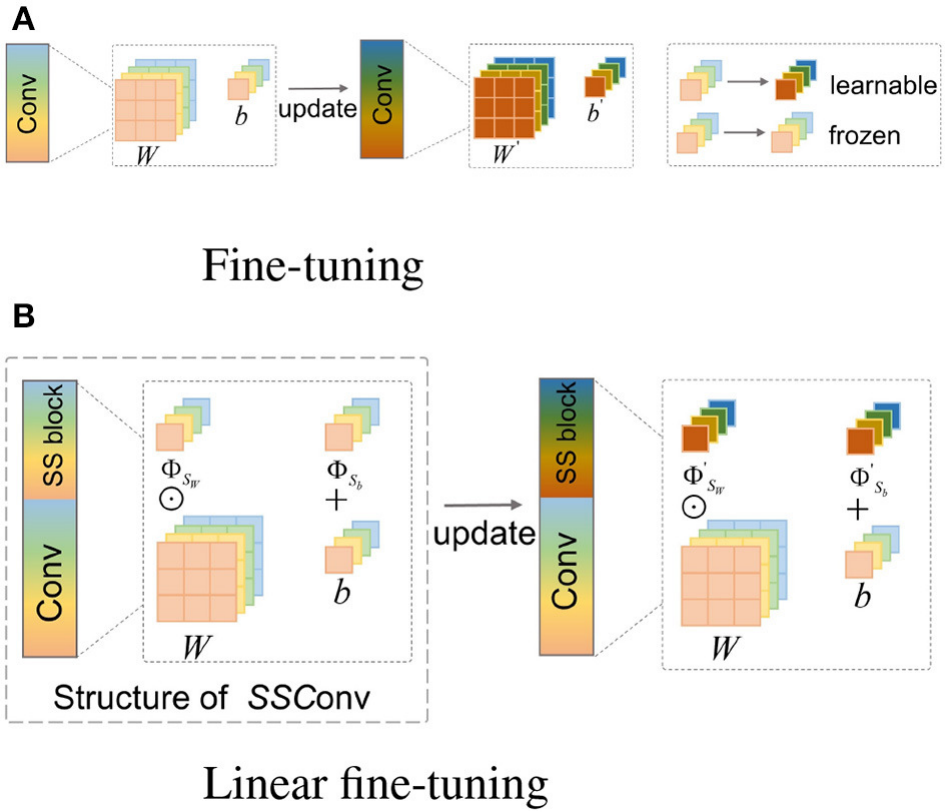
Visualization of the difference between FT and LFT in the transfer phase. Taking a 3 × 3 filter as an example with four channels, the structure of *SSConv* is composed of *SS* block and regular convolution. The 3 × 3 and 1 × 1 squares represent the weight *W* and bias *b* of the kernel, respectively, and different colors in them symbolize different channels. **(A)** FT updates all the pre-trained *W* and *b*. **(B)** The propose LFT optimizes the scaling factor Φ_*S*_*W*__ and shifting factor Φ_*S*_*b*__ only.

The aim for transfer learning is to generalize high-performance networks trained in a related source domain to the target domain (Weiss et al., 2016). Fine-tuning is a common transfer learning method for MRI reconstruction, which begins with pre-training a network in the source domain, usually with large-scale data. In the transfer phase, a new network is initialized with the pre-trained weights and continues to be trained for target task, with the updating process is shown in Figure 2A.

During pre-training, regular convolution *Conv* is applied to the input *U*^*s*^ in the source domain with a kernel *K*_(*m*)_ as


(4) $$\begin{array}{l} V_{b}^{s}=U^{s}⊙W+b. \end{array}$$

Note that *m* is omitted for readability. During the transfer process, FT initializes the target network with the pre-trained *W* and *b*, giving


(5) $$\begin{array}{l} V_{b}^{t}=U^{t}⊙W+b. \end{array}$$

Afterwards, FT updates all the values of *W* and *b* utilizing the target data *U*_*t*_, obtaining


(6) $$\begin{array}{l} V_{b}^{t}=U^{t}⊙W^{\prime }+b^{\prime }, \end{array}$$

where *W*′ and *b*′ indicate the updated weights.

Fine-tuning is considered to shorten the convergence time and enhance the quality of the generated images by providing a strong initialization, particularly when the target dataset is limited (Jiang et al., 2019). However, as stated in Section 1, such full-weight updating leads to “catastrophic forgetting” and overfitting.

### 2.2. Linear fine-tuning

#### 2.2.1. Hypothesis

Despite the presence of distinct variations in advanced features, they share many of the basic features (Olah et al., 2020). Hence, we hypothesize that for two similar types of images, their advanced features *V*_*a*_ can be derived from different linear combinations of the shared basic features *V*_*b*_ by means of the scaling factor Φ_*S*_*W*__ and shifting factor Φ_*S*_*b*__. By substituting the inputs in Equation (3) with data *U*^1^ and *U*^2^ from these two datasets, and adding two factors, the hypothesis can be expressed as


(7) $$\begin{array}{l} V_{a}^{1}=\sum_{m=1}^{M}\left(({\text{Φ}}_{S_{W}(m)}^{1}\cdot (U_{\left(m\right)}^{1}⊙W_{\left(m\right)})+({\text{Φ}}_{S_{b}(m)}^{1}+b_{\left(m\right)})\right),\\ V_{a}^{2}=\sum_{m=1}^{M}\left(({\text{Φ}}_{S_{W}(m)}^{2}\cdot (U_{\left(m\right)}^{2}⊙W_{\left(m\right)})+({\text{Φ}}_{S_{b}(m)}^{2}+b_{\left(m\right)})\right), \end{array}$$

where Φ_*S*_*W*_(*m*)_ and Φ_*S*_*b*_(*m*)_ denote the two factors corresponding to *K*_(*m*)_. Equation (7) indicates that *V*_*b*_ of *U*^1^ and *U*^2^ are extracted with the same kernel, and then their own *V*_*a*_ are formed by diverse linear combinations of *V*_*b*_, which are adjusted by *SS* factors. The above equation can be rearranged into


(8) $$\begin{array}{l} V_{a}^{1}=\sum_{m=1}^{M}\left((U_{\left(m\right)}^{1}⊙({\text{Φ}}_{S_{W}(m)}^{1}\cdot W_{\left(m\right)})+({\text{Φ}}_{S_{b}(m)}^{1}+b_{\left(m\right)})\right),\\ V_{a}^{2}=\sum_{m=1}^{M}\left((U_{\left(m\right)}^{2}⊙({\text{Φ}}_{S_{W}(m)}^{2}\cdot W_{\left(m\right)})+({\text{Φ}}_{S_{b}(m)}^{2}+b_{\left(m\right)})\right). \end{array}$$

Equation (8) indicates the effect of *SS* factors on the kernel. The Φ_*S*_*W*__ uniformly scales all the weights *W* of the kernel, while Φ_*S*_*b*__ shifts the outputs in addition to the original bias *b*. A linear transformation can be observed between the trained models for these two datasets.

Although the flexible sequences in MRI can result in images with varying advanced features, we argue that there is high repeatability of basic features due to the standardized views of medical images such as the limited texture variants or small patches (Alzubaidi et al., 2021). According to the hypothesis above, we speculate that there is a linear correlation relationship between the trained models for two related MR datasets. If these two are treated as source and target datasets separately, it is feasible to adjust the source model pre-trained on the source dataset, by *SS* factors to obtain the target model.

#### 2.2.2. Scaling and shifting factors

Based on the hypothesis and analysis, we propose the LFT strategy, which is implemented using the *SS* factors. The *SS* block, containing two factors, is integrated with regular convolution into *SSConv* in the transfer phase. The structure of *SSConv* is shown in Figure 2B. Taking a 3 × 3 filter as an example, the lower part of *SSConv* shows the regular convolution *Conv*. The upper part is *SS* block, which contains a single scaling factor Φ_*S*_*W*__ corresponding to *W* with 9 elements, and a shift factor Φ_*S*_*b*__ corresponding to *b*.

In the following, the complete training process of LFT is described in detail. Firstly, in the pre-training phase, the *W* and *b* are initialized randomly, which is identical to classic pre-training. We train the *W* and *b* on large-scale datasets, as represented by Equation (4). Next in the transfer phase, the new target model is initialized with the pre-trained weights, and the *SS* block is inserted into the model. Here Φ_*S*_*W*__ and Φ_*S*_*b*__ are initialized as 1 and 0, respectively. Therefore, feeding input *U*^*t*^ in the target domain into *SSConv* can be expressed as


(9) $$\begin{array}{l} V_{b}^{t}=U^{t}⊙\left({\text{Φ}}_{S_{W}}\cdot W\right)+\left({\text{Φ}}_{S_{b}}+b\right). \end{array}$$

Then LFT requires training the target model with target data, as shown in Figure 2B. It is worth noting that *W* and *b* are frozen, and only Φ_*S*_*W*__ and Φ_*S*_*b*__ are optimized, giving


(10) $$\begin{array}{l} V_{b}^{t}=U^{t}⊙\left({\text{Φ}}_{S_{W}}^{\prime }\cdot W\right)+\left({\text{Φ}}_{S_{b}}^{\prime }+b\right), \end{array}$$

where ${\text{Φ}}_{S_{W}}^{\prime }$ and ${\text{Φ}}_{S_{b}}^{\prime }$ indicate the optimized weights.

Figure 2 visualizes the difference between FT and LFT in the transfer phase. Fine-tuning updates the complete values of *W* and *b*, while LFT updates only the *SS* factors. It is obvious that LFT reduces the number of tuning weights, which contributes to lessening the risk of overfitting in the case of small samples sizes. Moreover, LFT prevents the problem of “catastrophic forgetting” by fixing the pre-trained weights, which benefits in learning specific knowledge of the target data without forgetting the generic knowledge learned from the source data.

### 2.3. Materials

#### 2.3.1. Network

**Figure 3:**
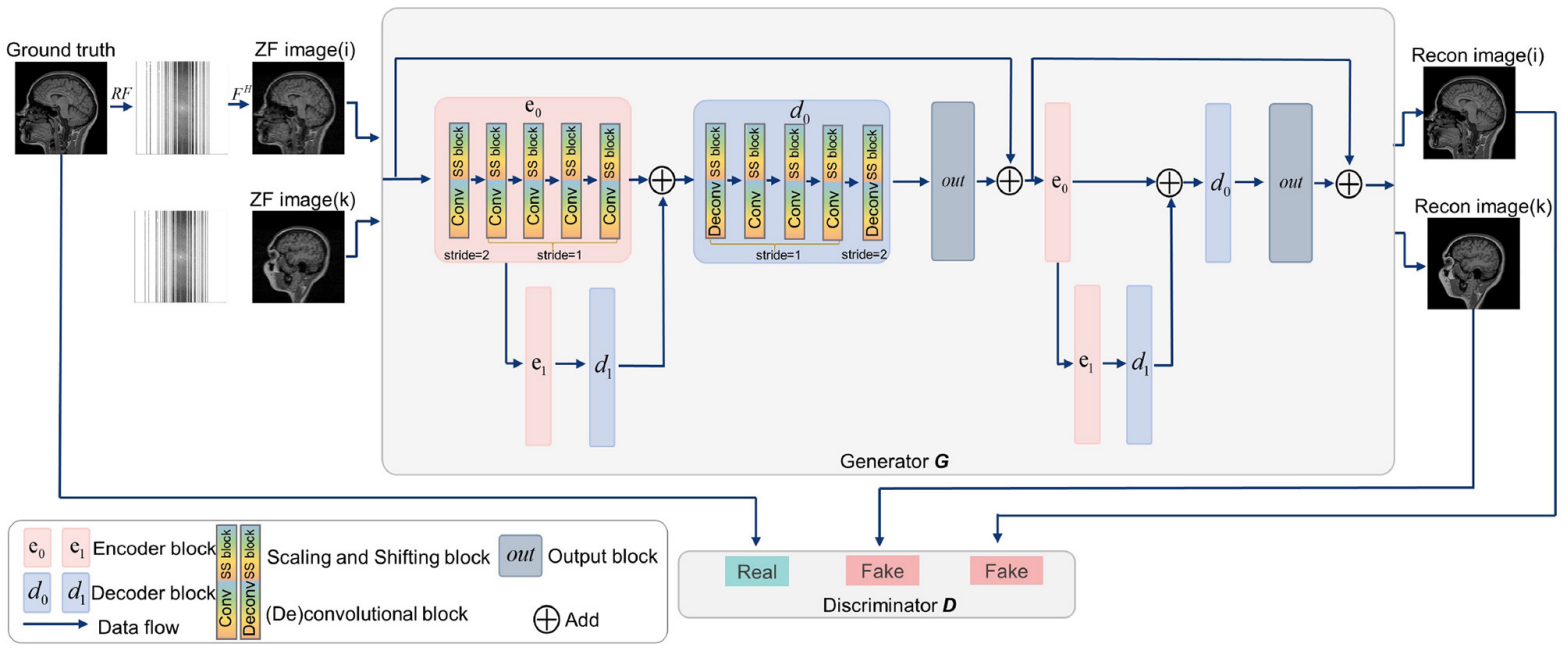
Overview of the SSGAN architecture. The generator *G* of SSGAN is composed of two residual U-net with 2 encoder (pink box) and 2 decoder (blue box) blocks. The architecture of the discriminator *D* is the same as the encoding path of *G*. The inputs of *G* are the ZF image (i) and ZF image (k), which come from different collections.

Generative adversarial networks (GANs) have shown strong performance in modeling the prior distributions of images (Shaul et al., 2020; Lv et al., 2021b). Therefore, GANs have been widely studied in MRI reconstruction (Shitrit and Riklin Raviv, 2017; Yang et al., 2017; Mardani et al., 2018; Quan et al., 2018), among which, RefineGAN (Quan et al., 2018) gets a superior performance (Lv et al., 2021b). In view of this, we constructed SSGAN as the reconstruction network by referring to RefineGAN's residual bottleneck block and double chain structure. To evaluate the proposed LFT strategy, the *SS* block is inserted into the network in the transfer phase. In addition, our network only contains basic building units, such as convolutional layers, non-linear activation functions and residual connections to facilitate the experiments on generalization and transferability. Figure 3 provides an overview of the SSGAN architecture with the inserted *SS* block. For more details, please refer to RefineGAN for more details.

#### 2.3.2. Datasets

The IXI dataset^1^ is a large publicly available MR brain image dataset of healthy subjects, and the extensive data assists in improving the performance of the original model. For pre-training the source model, 58,000 sagittal T1-weighted brain images were selected from the IXI dataset. The transfer performance of the source model was explored on three different target datasets: the private sagittal T2-weighted brain dataset I, the private axial T1-weighted brain dataset II and the FastMRI^2^ knee dataset. These datasets differ from the source data in distributions ranging from small to large, allowing us to study the effects of the distribution deviation on the proposed method. The two private datasets were derived from the study (Jiang et al., 2022), and all the ethical and experimental procedures were approved by the First Affiliated Hospital of University of Science and Technology of China (in accordance with the Declaration of Helsinki), under Application No. 2021 KY205. Detailed acquisition parameters can be found in the Supplementary material. Slices (256 × 256 pixels) were extracted from raw data as the standard reference images, and retrospective under-sampling was performed with the mask to obtain the under-sampled data. The mask was constructed by random row (1D) sampling from a normal distribution, with denser sampling in the central part of the k-space.

#### 2.3.3. Performance evaluation

The results were evaluated by the Peak Signal-to-Noise Ratio (PSNR) and the Structure SIMilarity (SSIM) (Wang et al., 2004) as they are commonly used measures for evaluating image reconstruction quality. Both metrics rely on a pixel-wise comparison between the fully-sampled image and the reconstructed result. In addition, weighted peak signal-to-noise ratio (WPSNR) (Gupta and Aggarwal, 2009; Erfurt et al., 2019) is considered to be a metric more compatible with human visual perception, and its results are included in the Supplementary material.

## 3. Experiment and results

### 3.1. Experimental design

**Figure 4:**
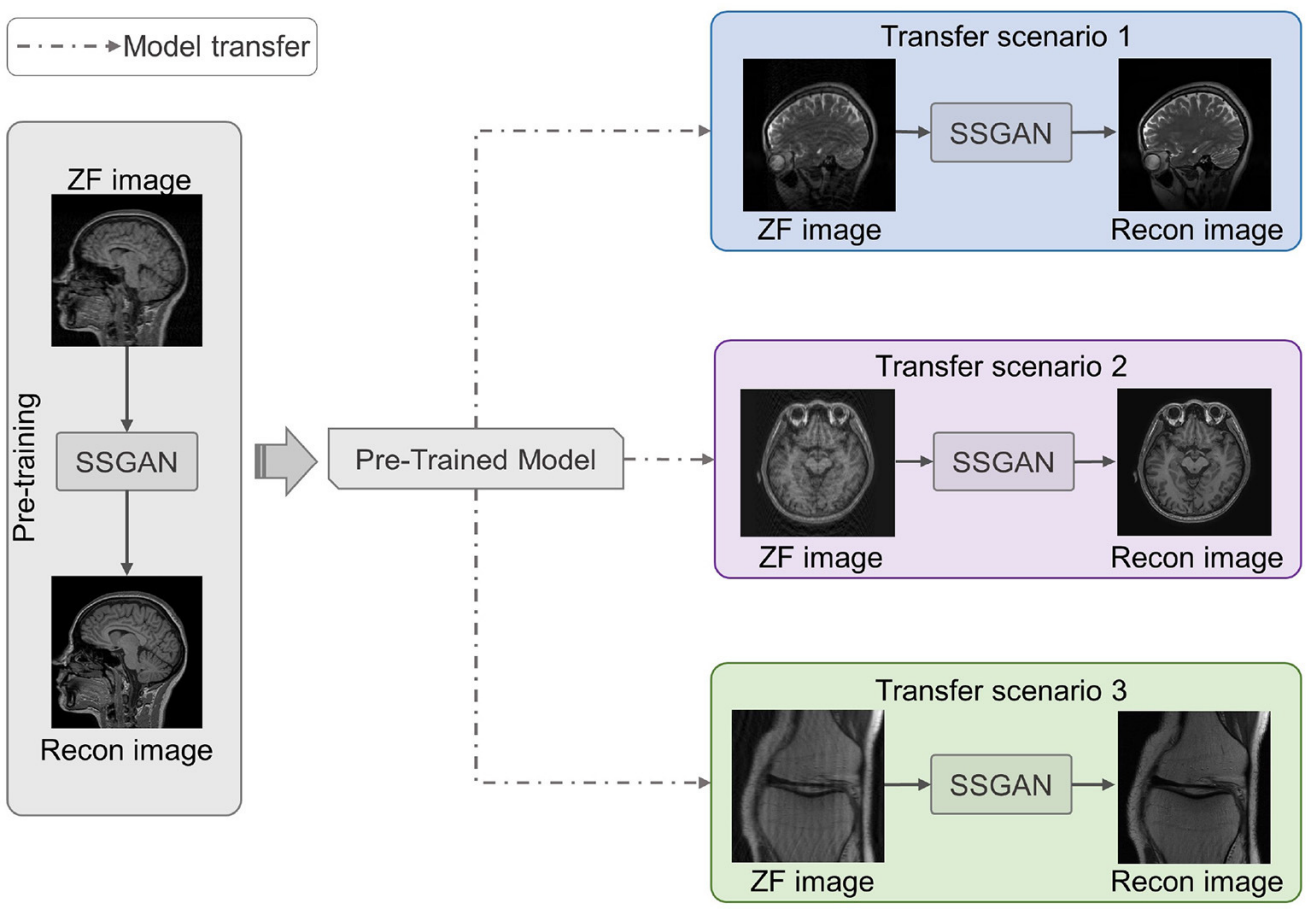
Three transfer scenarios of the pre-trained SSGAN. The gray part shows SSGAN was pre-trained on the IXI dataset, then transferred to reconstruct images with different contrasts (blue part, scenario 1: T1-weighted to T2-weighted), slicing directions (purple part, scenario 2: axial planes to sagittal planes), and anatomical structures (green part, scenario 3: brain to knee).

As shown in Figure 4, we utilized LFT to transfer the pre-trained SSGAN (gray part) to reconstruct the under-sampled MR data from three target datasets mentioned in II.D (color part). Hence, three transfer scenarios were designed: (1) sagittal brain MR images with different contrasts (T1-weighted and T2-weighted); (2) brain MR images with different slicing directions (axial and sagittal planes); (3) MR images with different anatomical structures (brain and knee).

The model transferred by LFT strategy was termed linear fine-tuning model (LFT model). For comparison, the following models were set up: (1) testing the target data directly with pre-trained model (PT model) to verify the necessity of transfer learning; (2) training the model directly (DT model) from scratch with the target data to prove the effectiveness of transfer learning; (3) transferring the PT model by FT, termed fine-tuning model (FT model).

Additionally, experiments were conducted at different sampling rates of 30, 40, and 50% to examine the robustness of the proposed method. We also investigated the effect of different sizes of training sets to validate the feasibility of LFT in the case of few samples.

### 3.2. Implementation details

All the models in this work were implemented using PyTorch framework on NVIDIA Geforce GTX 3090 with 24 GB memory. Adam optimizer (Kingma and Ba, 2014) with an initial learning rate of 10^−4^ was applied for pre-training, and lowered the initial learning rate depending on the target datasets in the transfer phase. We stopped the network training based on the convergence criterion that the PSNR on the validation set does not increase within 15 epochs. The datasets used for the experiments were divided into training, validation and test datasets in the ratio of 16:5:4, but not all in the training set were used every time to examine the effect of the training set size.

### 3.3. Validation of the hypothesis

**Figure 5:**
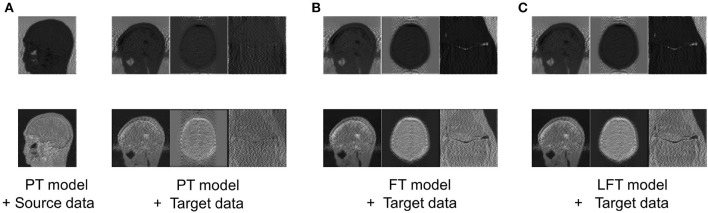
Comparison of advanced features extracted by filters of the same channel in four cases: advanced features obtained by **(A)** feeding the source data into the PT model; **(B)** feeding the target data into the PT model; **(C)** feeding the target data into the FT model; **(D)** feeding the target data into the LFT model. Source data is from IXI dataset, while target data are from private sagittal brain dataset I, private axial brain dataset II, and FastMRI knee dataset.

To verify the hypothesis in Section 2.2, the feature maps, i.e., advanced features extracted by filters of the same channel for four cases are visualized in Figure 5, including: advanced features obtained by (a) feeding the source data into the PT model; (b) feeding the target data into the PT model; (c) feeding the target data into the FT model; (d) feeding the target data into the LFT model.

As shown in column (a), the PT model extracted the out-of-contour artifacts (the first row) and in-contour artifacts (the last row) of source data, which presented the functions of these two pre-trained filters, respectively. The features in column (b) were the results of the PT model tested directly on the target data and were of different types than that in column (a) obviously. We attribute this to the fact that although the data in the source and target domains have similar basic features, the combination coefficients of advanced features are variable. Inappropriate coefficients prevented the PT model from extracting the expected advanced features of the target data. Column (c) shows the FT model restored the filters to extract the expected features, as in column (a). Although FT adjusted all the weights, it served to restore the original function of the filter, instead of relearning to extract new types. Column (d) displays the features of applying the LFT model to the target data. It is found that it also succeeded in extracting the desired advanced feature types. In particular, the LFT model adjusted the suitable coefficients for linear combinations of basic features by *SS* factors. This demonstrates that the hypothesis is reasonable and the LFT can achieve model transfer as FT with fewer tuning weights.

### 3.4. Results and analysis

In the pre-training phase, we trained three SSGANs with different under-sampling rates on the large-scale source IXI dataset. The networks reached the convergence stage within a few dozen iterations and achieved satisfactory reconstructions on the test data. Table 1 presents the quantitative evaluation of both PSNR and SSIM, where the values of PSNR and SSIM are displayed in the form of mean ± standard deviation. The zero-filled model (ZF model) indicates the zero-filled reconstruction of the under-sampled k-space measurement. Each network with the highest PSNR was saved as the basis for further transfer and named as PT model.

**Table 1:** Performance evaluation of PT models on source dataset.

**PSNR/SSIM**	**30%**		**40%**		**50%**	
**model**	**PSNR**	**SSIM**	**PSNR**	**SSIM**	**PSNR**	**SSIM**
ZF model^*a*^	25.78 ± 2.35	0.691 ± 0.046	26.17 ± 2.32	0.696 ± 0.046	28.72 ± 2.23	0.745 ± 0.046
PT model^*b*^	39.02 ± 2.81	0.981 ± 0.008	39.83 ± 3.12	0.982 ± 0.008	42.24 ± 3.15	0.988 ± 0.005

^*a*^Zero-filled model. ^*b*^Pre-trained model.

**Figure 6:**
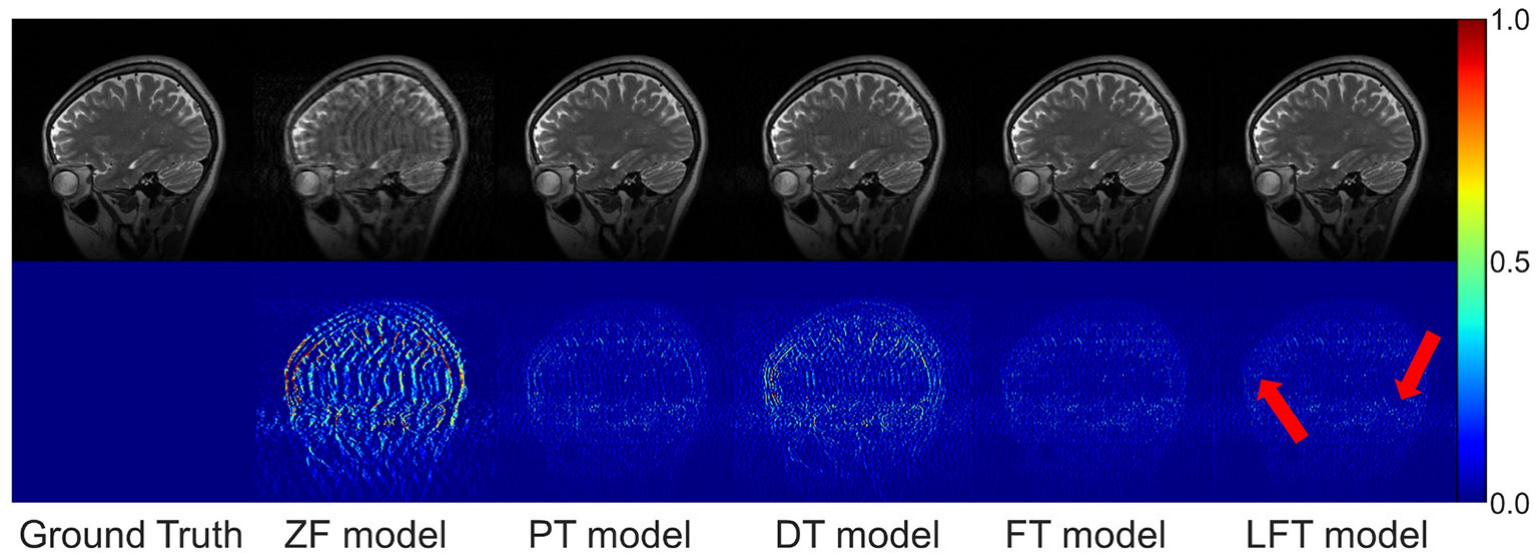
Typical reconstructions for sagittal T2-weighted brains from private dataset I by different methods. The last three models trained with 160 images at 50% sampling rate. From left to right are the results of: ground truth, zero-filled model, pre-trained model, directly trained model, fine-tuning model, and linear fine-tuning model, as well as their 10× magnified error maps.

In transfer scenario 1, the target data are sagittal T2-weighted brain images, with different contrasts from the source data. There is not much deviation between their distributions. Table 2 demonstrates the evaluation metrics of different reconstructions for the target data, with bolded indicating the best. The mean SSIM and PSNR values were both improved for two transfer models compared to the original PT and DT model. It indicates the necessity and effectiveness of transfer learning in the presence of contrast variation between the source and target data. In addition, the highest PSNR and SSIM were obtained by the LFT model at various under-sampling rates, reflecting the superiority and robustness of the proposed method. Figure 6 provides the reconstructions of each model. It can be seen that the reconstruction of the DT model had notable artifacts, indicating that training the network from scratch could not achieve a good result when there were only a few target samples. Instead, PT, FT, and LFT models basically succeeded in reconstructing, but the results of the LFT model achieved artifact minimization, which is more obvious at the red arrow indication in the error maps. Therefore, LFT model provided the most desirable reconstruction when transferring the network to data with different contrasts.

**Table 2:** Performance evaluation of different reconstructions for the target data with different contrasts.

**PSNR/SSIM**	**30%**		**40%**		**50%**	
**model**	**PSNR**	**SSIM**	**PSNR**	**SSIM**	**PSNR**	**SSIM**
ZF model^*a*^	31.10 ± 2.73	0.850 ± 0.048	31.90 ± 2.92	0.853 ± 0.049	33.88 ± 2.76	0.891 ± 0.038
PT model^*b*^	40.45 ± 2.39	0.973 ± 0.007	41.28 ± 2.56	0.978 ± 0.008	44.52 ± 2.61	0.980 ± 0.003
DT model^*c*^	37.45 ± 2.61	0.959 ± 0.013	37.43 ± 2.77	0.956 ± 0.015	39.82 ± 2.60	0.971 ± 0.010
FT model^*d*^	40.72 ± 2.54	0.981 ± 0.008	42.35 ± 2.73	0.984 ± 0.008	44.78 ± 2.69	0.991 ± 0.004
LFT model^*e*^	**41.45** **±2.52**	**0.984** **±0.008**	**42.52** **±2.78**	**0.985** **±0.008**	**45.84** **±2.64**	**0.992** **±0.003**

^*a*^Zero-filled model. ^*b*^Pre-trained model. ^*c*^Directly trained model. ^*d*^Fine-tuning model. ^*e*^Linear fine-tuning model. The bold values indicate the best values of the evaluation metrics of different reconstructions for the target data.

**Figure 7:**
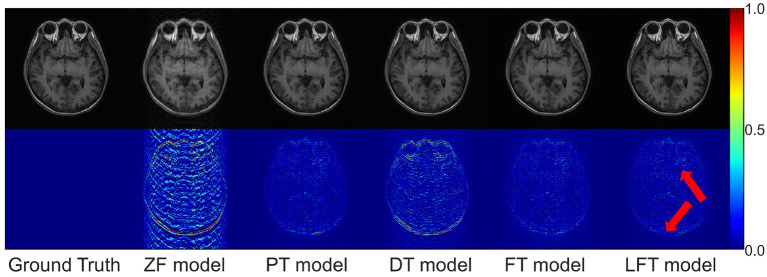
Typical reconstructions for axial T1-weighted brain images from private dataset II by different models (the last three networks trained with 200 images at 50% sampling rate). From left to right are the results of: ground truth, zero-filled model, pre-trained model, directly trained model, fine-tuning model, and linear fine-tuning model.

As for transfer scenario 2, the target data are axial T1-weighted brain images. They are sliced in different directions from the source data, and the variance in the data distribution leads to some respective features. Table 3 shows the quantitative results of different reconstructions on the target data. The bolded rows mark the best results obtained by the LFT model, resolving the variation in different slicing directions. It is noteworthy that the LFT model demonstrated high reconstruction quality for small training sets, while the FT model showed a clear performance degradation as the sample size decreased. As shown in the results of the 100 training images, the LFT model reflects the most visible advantage over the FT model, with a 2.06 dB (5.89%) improvement in PSNR. Figure 7 presents intuitive reconstructions for the target data. A slight difference can be observed from the red arrow indication in the error map, which represents the LFT model outperforming the others in scenario 2.

**Table 3:** Performance evaluation of different reconstructions for the target data with different slicing directions at 30% sampling rate.

**PSNR/SSIM**	**100 images**		**200 images**		**400 images**		**800 images**	
**model**	**PSNR**	**SSIM**	**PSNR**	**SSIM**	**PSNR**	**SSIM**	**PSNR**	**SSIM**
ZF model^*a*^	23.88 ± 3.47	0.806 ± 0.040	23.88 ± 3.47	0.806 ± 0.040	23.88 ± 3.47	0.806 ± 0.040	23.88 ± 3.47	0.806 ± 0.040
PT model^*b*^	34.33 ± 2.67	0.959 ± 0.009	34.33 ± 2.67	0.959 ± 0.009	34.33 ± 2.67	0.959 ± 0.009	34.33 ± 2.67	0.959 ± 0.009
DT model^*c*^	31.46 ± 2.84	0.925 ± 0.016	32.62 ± 2.87	0.941 ± 0.014	33.92 ± 2.77	0.958 ± 0.011	34.86 ± 2.71	0.964 ± 0.011
FT model^*d*^	34.94 ± 2.61	0.950 ± 0.026	35.05 ± 2.61	0.951 ± 0.027	35.21 ± 2.61	0.952 ± 0.027	35.40 ± 2.56	0.950 ± 0.030
RFT model^*e*^	36.82 ± 2.51	0.952 ± 0.042	36.88 ± 2.48	0.952 ± 0.047	36.95 ± 2.45	0.954 ± 0.051	37.00 ± 2.49	0.955 ± 0.050
LFT model^*f*^	**37.00** **±1.89**	**0.966** **±0.012**	**37.03** **±1.90**	**0.966** **±0.015**	**37.13** **±1.97**	**0.968** **±0.018**	**37.14** **±1.93**	**0.968** **±0.013**

^*a*^Zero-filled model. ^*b*^Pre-trained model. ^*c*^Directly trained model. ^*d*^Fine-tuning model. ^*e*^Row fine-tuning model. ^*f*^Linear fine-tuning model. The bold values indicate the best values of the evaluation metrics of different reconstructions for the target data.

Furthermore, we consider FT as a way for each element in a kernel to learn a transformation factor, and LFT as a way for all elements in a kernel to learn a transformation factor. Therefore, the method of learning one transformation factor for all elements in a single row within the kernel is set up for comparison, named row fine-tuning (RFT) model. The results of RFT were added to Table 3 as a comparison, and we can observe that RFT improves upon FT by reducing adjustment weights. However, LFT, which minimizes the number of updated weights, yields the best performance. It implies that the performance of the transfer can be improved by reducing the number of weight updates, and LFT proves to be the optimal choice.

**Figure 8:**
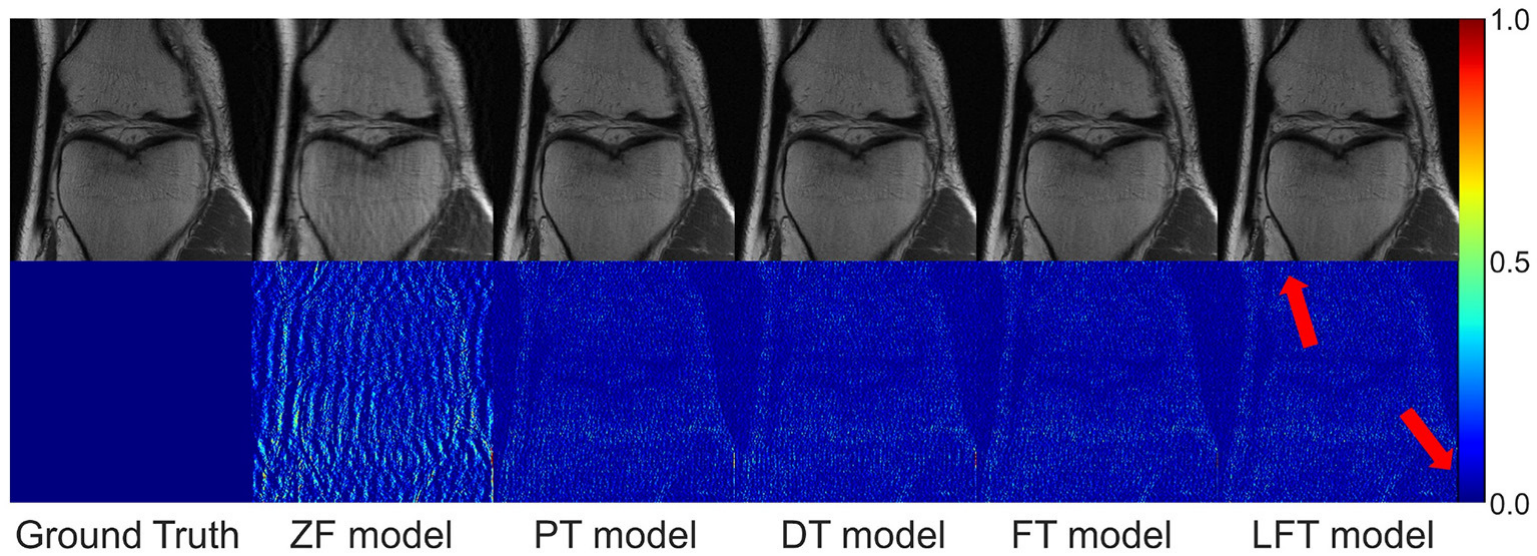
Typical reconstructions for knee images from FastMRI dataset by different networks (the last three networks trained with 200 images at 50% sampling rate). From left to right are the results of: ground truth, zero-filled model, pre-trained model, directly trained model, fine-tuning model, and linear fine-tuning model.

We transferred the model pre-trained on the brain data to reconstruct knee images in scenario 3, which belong to different anatomical structures and vary greatly from the source data. The reconstruction indices for the knee data at 50% sampling rate are presented in Table 4. The results indicate that LFT model obtained the optimal quality in most cases, but occasionally, the FT model performed better. We consider this phenomenon reasonable due to the large variation in diverse anatomical structures. Relatively few similar features limited the validity of LFT method. However, FT adjusted the model more adequately after providing more training data, resulting in better performance. It is also reflects by the decreasing gap between the metrics of FT and LFT models as the number of training sets increased. Besides, both FT and LFT models deteriorated the PT model due to overfitting when the dataset was extremely small. As the training data increased, LTF prioritized improving the situation. Typical reconstructions for knee images by different networks are shown in Figure 8. Focusing on the error maps of the PT model here, there were more artifacts remaining in the background compared to the first two scenarios. This observation suggests that the PT model was more adaptable to the first two datasets as the data are more similar. In addition, the red arrow indicates that the LFT model had the least residual artifacts in the reconstruction images. Consequently, the LFT method is still the optimal transfer strategy when the source and limited target data vary widely.

**Table 4:** Performance evaluation of different reconstructions for the target data with different anatomical structures at 50% sampling rate.

**PSNR/SSIM**	**100 images**		**200 images**		**400 images**		**800 images**	
**model**	**PSNR**	**SSIM**	**PSNR**	**SSIM**	**PSNR**	**SSIM**	**PSNR**	**SSIM**
ZF model^*a*^	29.34 ± 3.07	0.854 ± 0.038	29.34 ± 3.07	0.854 ± 0.038	29.34 ± 3.07	0.854 ± 0.038	29.34 ± 3.07	0.854 ± 0.038
PT model^*b*^	35.35 ± 2.86	**0.934** **±0.025**	35.35 ± 2.86	0.934 ± 0.025	35.35 ± 2.86	0.934 ± 0.025	35.35 ± 2.86	0.934 ± 0.025
DT model^*c*^	32.51 ± 2.86	0.907 ± 0.024	33.92 ± 2.75	0.922 ± 0.021	34.76 ± 2.64	0.930 ± 0.020	35.14 ± 2.59	0.934 ± 0.018
FT model^*d*^	34.70 ± 2.69	0.930 ± 0.024	35.26 ± 2.76	0.933 ± 0.024	35.50 ± 2.79	**0.936** **±0.024**	35.86 ± 2.72	**0.936** **±0.024**
LFT model^*e*^	**35.64** **±2.33**	0.932 ± 0.024	**35.79** **±2.55**	**0.934** **±0.024**	**35.83** **±2.60**	0.934 ± 0.024	**35.96** **±2.66**	0.935 ± 0.024

^*a*^Zero-filled model. ^*b*^Pre-trained model. ^*c*^Directly trained model. ^*d*^Fine-tuning model. ^*e*^Linear fine-tuning model. The bold values indicate the best values of the evaluation metrics of different reconstructions for the target data.

## 4. Discussion

This study centers on optimizing the FT strategy for MRI reconstruction. We conducted a comparative analysis of FT, LFT, and other transfer strategies in various transfer scenarios based on the reconstruction quality. Fine-tuning yields suboptimal reconstruction quality and appreciable residual artifacts, especially when the target domain training set contains fewer than 200 images in the anatomical structure transfer scenario, whereby the FT model performs worse than the PT model. We attribute the result to “catastrophic forgetting” and overfitting. Fine-tuning involves the update of all weights, which inevitably forgets the pre-trained knowledge. Moreover, as the target domain typically has limited data, updating a large number of weights is prone to overfitting (Sun et al., 2019). Obviously, reducing the number of updated weights is crucial to improve the FT's performance. Several studies (Tajbakhsh et al., 2016; Amiri et al., 2020) have attempted to update only a subset of network weights, but the required level of tuning differs from one application to another. Hence, this approach is only applicable to certain transfer scenarios, limiting its scalability. The proposed LFT provides a new perspective of decoupling the pre-trained and to-be-updated weights by introducing learnable *SS* factors. While completely fixing the pre-trained weights, LFT can accomplish the transfer task by updating the weights fewer than those in the FT, thereby avoiding forgetting and overfitting. Consequently, LFT achieves more competitive results in various transfer scenarios.

Comprehensively analyzing the results of multiple transfer scenarios, it is found that with only 100 training images in the target domain, LFT outperforms FT with 400 training images. This implies that in the practical deployment, LFT can construct multiple reconstruction models at a lower cost and shorter development cycles to adapt to various complicated clinical scenarios. In addition, the *SS* block can be integrated into existing convolutional neural networks in a plug-and-play fashion, further enhancing the clinical applicability of LFT.

This study has some limitations. Firstly, we have exclusively assessed the proposed method using the linear sampling pattern of the Cartesian k-space trajectory, owing to its prevalence. Additional research is necessary to examine the feasibility of utilizing LFT in non-linear scenarios, such as radial sampling patterns. Secondly, deep MRI reconstruction is performed using retrospectively under-sampled data, which deviates from clinical routine, so prospective validation is required. Thirdly, LFT introduces a few additional parameters to the original model, causing an increase in the latency of inference. The comparison of inference time with and without additional parameters is shown in the Supplementary material. Despite a slight extension of reconstruction time, it does not considerably affect the overall MRI process in most clinical applications, as data acquisition and reconstruction can be executed asynchronously. Lastly, our source domain dataset only contains brain images, that is, the transfer effects were verified only from brain MR images to other scenarios. In future work, we intend to gather a more diverse range of image types to thoroughly evaluate the transferability of the LFT strategy across various scenarios.

## 5. Conclusion

To address the issues of “catastrophic forgetting” and overfitting in FT for MRI reconstruction, we have developed a novel transfer strategy, LFT, which is predicated on a linear transformation hypothesis. By focusing on optimizing the *SS* factors, as opposed to all weights, LFT achieves performance on par with FT, while requiring fewer training samples from the target domain. When applying deep learning for MRI reconstruction in diverse and complicated clinical scenarios, engineers only need to create a general pre-training model using MR images from various sequences and body parts, and subsequently gather a small quantity of images in the target scene. The LFT approach can then be employed to derive a customized reconstruction model with satisfactory performance. Additionally, as the LFT method can be seamlessly integrated with any reconstruction convolutional network, it does not limit the choice of architecture during the development phase. To conclude, LFT greatly enhances the viability of deep MRI reconstruction in scenarios with limited data.

## Data availability statement

Publicly available datasets were analyzed in this study. This data can be found at: http://brain-development.org/ixi-dataset/ and https://fastmri.org/dataset.

## Ethics statement

The studies involving human participants were reviewed and approved by the First Affiliated Hospital of University of Science and Technology of China. No. 2021 KY205. The patients/participants provided their written informed consent to participate in this study.

## Author contributions

WB: methodology, conducting the experiments, and writing—original draft preparation. JX: implementing the algorithm. MS: data curation. XH: methodology, supervision, and writing—review and editing. DG: guidance and supervision. FQ: methodology and writing—review and editing. All authors contributed to the article and approved the submitted version.

## Footnotes

^1^ http://brain-development.org/ixi-dataset/

^2^ https://fastmri.org/dataset/
