# Supplementary material for: Linear fine-tuning: a linear transformation based transfer strategy for deep MRI reconstruction
Source: Front Neurosci. 2023 Jun 20;17:1202143. doi: 10.3389/fnins.2023.1202143 (PMC10318193; doi:10.3389/fnins.2023.1202143)
Supplement: Supplementary file 1 [file Data_Sheet_1.PDF]

Supplementary Material

1 SUPPLEMENTARY DATA

1.1 Acquisition parameters

Both private datasets were collected from 3T scanners (GE MEDICAL SYSTEM– DISCOVER MR750). Private sagittal brain dataset I comprises a total of 1980 sagittal T2-weighted brain images from nine healthy subjects, which is used to explore the transfer performance of the model from source data to images with different contrasts. These images were acquired using Sag CUBE T2 sequence with the following parameters: TR=2500ms, TE=81.11ms. The resolution of the images from dataset I is $320 \times 320 \times 220$; Private axial brain dataset II consists of 2500 axial T1-weighted brain images, gathered to investigate the model's performance when reconstructing images with different slicing directions. The sequence 3D T1-BRAVO used in Private axial brain dataset II was adopted with the following parameters: TR = 8.2ms, TE = 3.2ms, TI = 1.0, and FOV = 24×24. The resolution of the images from dataset II is $256 \times 256 \times 188$; FastMRI dataset, a large-scale public collection of MR raw measurements and clinical images, was used in our study. From this dataset, 2500 coronal proton density-weighted knee images were randomly selected for analyzing the results in scenarios involving transfer across different anatomical structures.

1.2 Weighted peak signal-to-noise ratio

Weighted peak signal-to-noise ratio: WPSNR(Erfurt et al., 2019; Gupta and Aggarwal, 2009) is an image quality assessment metric similar to PSNR, but it takes into account the importance of different regions. WPSNR is more compatible with eye perception than the standard PSNR. Therefore, the results of WPSNR are added to supplement the experiments.

The formula for calculating WPSNR is as follows:

$$WPSNR = 10 \cdot \log_{10} \left[\mathrm{L}^2 / \left(\sum w \cdot (\mathrm{u} - \mathrm{v})^2 \right) \right], \tag{S1}$$

where L is the maximum possible range of pixel values, w denotes the weight of each pixel, and u and v denote the grayscale values of the corresponding pixels in the original and compressed images, respectively. To determine the weight of each pixel, a common method is to smooth the original image using a Gaussian kernel. The smoothed image pixels are compared with the original image to obtain a weight matrix.

Table S1S2S3 presents the WPSNR of the reconstruction results from each model for the target data in different transfer scenarios.

Table S1. Performance evaluation of different reconstructions for the target data with different contrasts.

Model	30% WPSNR	40% WPSNR	50% WPSNR
ZF model[a]	31.01±2.73	31.97±2.95	33.82±2.77
PT model[b]	39.97±2.39	41.03±2.57	44.31±2.66
DT model[c]	36.93±2.55	37.10±2.72	39.41±2.55
FT model[d]	40.26±2.55	42.20±2.74	44.52±2.74
LFT model[e]	**40.98±2.53**	**42.08±2.80**	**45.68±2.70**

Note: [a] Zero-filled model. [b] Pre-trained model. [c] Directly trained model. [d] Fine-tuning model. [e] Linear fine-tuning model.

Table S2. Performance evaluation of different reconstructions for the target data with different slicing directions at 30% sampling rate.

Model	100 images WPSNR	200images WPSNR	400 images WPSNR	800 images WPSNR
ZF model[a]	23.87±3.50	23.87±3.50	23.87±3.50	23.87±3.50
PT model[b]	33.82±2.68	33.82±2.68	33.82±2.68	33.82±2.68
DT model[c]	31.13±2.80	32.21±2.85	33.46±2.77	34.36±2.70
FT model[d]	34.46±2.62	34.56±2.62	34.71±2.61	34.89±2.56
LFT model[e]	**36.41±1.91**	**36.43±1.90**	**36.54±1.98**	**36.59±1.94**

Note: [a] Zero-filled model. [b] Pre-trained model. [c] Directly trained model. [d] Fine-tuning model. [e] Linear fine-tuning model.

Table S3. Performance evaluation of different reconstructions for the target data with different anatomical structures at 50% sampling rate.

Model	100 images WPSNR	200images WPSNR	400 images WPSNR	800 images WPSNR
ZF model[a]	29.65±3.09	29.65±3.09	29.65±3.09	29.65±3.09
PT model[b]	35.46±2.89	35.46±2.89	35.46±2.89	35.46±2.89
DT model[c]	32.65±2.89	34.03±2.77	34.85±2.67	35.23±2.61
FT model[d]	34.75±2.68	35.30±2.76	35.53±2.77	35.78±2.71
LFT model[e]	**35.61±2.42**	**35.78±2.55**	**35.83±2.59**	**35.97±2.64**

Note: [a] Zero-filled model. [b] Pre-trained model. [c] Directly trained model. [d] Fine-tuning model. [e] Linear fine-tuning model.

1.3 Comparison of inference time

We conducted a time study about the latency of inference in three transfer scenarios. The inference time of models were calculated with and without additional parameters for each image. The results are shown in Table S4.

Table S4. Comparison of inference time with and without additional parameters.

Inference time (s)	FT model[a]	LFT model[b]
Transfer scenario 1	0.01658	0.01707
Transfer scenario 2	0.01798	0.01898
Transfer scenario 3	0.05032	0.05236

Note: [a] Fine-tuning model (without additional parameters). [b] Linear fine-tuning model (with additional parameters).

REFERENCES

Erfurt, J., Helmrich, C. R., Bosse, S., Schwarz, H., Marpe, D., and Wiegand, T. (2019). A study of the perceptually weighted peak signal-to-noise ratio (wpsnr) for image compression. In *2019 IEEE International Conference on Image Processing (ICIP)* (IEEE), 2339–2343

Gupta, G. and Aggarwal, H. (2009). Digital image watermarking using two dimensional discrete wavelet transform, discrete cosine transform and fast fourier transform. *International Journal of Recent Trends in Engineering* 1, 616
